# *NRT1.1B* mediates rice plant growth and soil microbial diversity under different nitrogen conditions

**DOI:** 10.1186/s13568-024-01683-7

**Published:** 2024-04-22

**Authors:** Yawen Ju, Yanyan Jia, Baoshan Cheng, Di Wang, Dalu Gu, Wenjiang Jing, Hao Zhang, Xinhong Chen, Gang Li

**Affiliations:** 1 Huai’an Key Laboratory of Agricultural Biotechnology, Huaiyin Institute of Agricultural Science in Xuhuai Region of Jiangsu, Huai’an, 223001 China; 2 Jiangsu Key Laboratory of Crop Genetics and Physiology, Jiangsu Key Laboratory of Crop Cultivation and Physiology, Jiangsu Co-Innovation Center for Modern Production Technology of Grain Crops, Yangzhou University, Yangzhou, 225009 China; 3 School of Life Science and Food Engineering, Huaiyin Institute of Technology, Huai’an, 223001 China

**Keywords:** Bacterial diversity, Nitrogen fertilizer, *NRT1.1B* gene, Plant growth, Rice variety

## Abstract

Interactions between microorganisms and plants can stimulate plant growth and promote nitrogen cycling. Nitrogen fertilizers are routinely used in agriculture to improve crop growth and yield; however, poor use efficiency impairs the optimal utilization of such fertilizers. Differences in the microbial diversity and plant growth of rice soil under different nitrogen application conditions and the expression of nitrogen-use efficiency-related genes have not been previously investigated. Therefore, this study investigates how nitrogen application and nitrogen-use efficiency-related gene *NRT1.1B* expression affect the soil microbial diversity and growth indices of two rice varieties, *Huaidao 5* and *Xinhuai 5*. In total, 103,463 and 98,427 operational taxonomic units were detected in the soils of the *Huaidao* 5 and *Xinhuai* 5 rice varieties, respectively. The Shannon and Simpson indices initially increased and then decreased, whereas the Chao and abundance-based coverage estimator indices decreased after the application of nitrogen fertilizer. Nitrogen fertilization also reduced soil bacterial diversity and richness, as indicated by the reduced abundances of *Azotobacter* recorded in the soils of both rice varieties. Nitrogen application initially increased and then decreased the grain number per panicle, yield per plant, root, stem, and leaf nitrogen, total nitrogen content, glutamine synthetase, nitrate reductase, urease, and root activities of both varieties. Plant height showed positive linear trends in response to nitrogen application, whereas thousand-grain weights showed a negative trend. Our findings may be used to optimize nitrogen fertilizer use for rice cultivation and develop crop-variety-specific strategies for nitrogen fertilizer application.

**Supplementary Information:**

The online version contains supplementary material available at 10.1186/s13568-024-01683-7.

## Introduction

Rice (*Oryza sativa* L.) is the most important cereal food crop in China, playing a vital role in the country’s food security and economic development. The *indica* and *japonica* varieties are the predominantly cultivated rice varieties in Asia (Wang et al. 2018b). In the field, the *japonica* varieties have lower nitrogen-use efficiency than the *indica* varieties, which results in lower yield and limits their widespread cultivation (Rakotoson et al. 2017; Xu et al. 2023). Nitrogen is essential for the growth and development of plants, and it is used at all growth stages, including the tillering, flowering, and mature stages in rice, thereby directly influencing crop growth and development (Ladha et al. 2022). The application of nitrogen fertilizers in rice production to increase productivity is becoming a common practice for increasing crop productivity. However, the application of a high quantities of nitrogen fertilizer not only reduces nitrogen-use efficiency but also exacerbates environmental problems, such as resource waste and water eutrophication (Yang and Zhang 2023). An effective strategy for improving rice nitrogen-use efficiency of rice is to fully exploit the potential of existing rice germplasm resources to absorb and use nitrogen more effectively. Thus, introducing the rate-related high-nitrogen-use *indica* gene *NRT1.1B* in *japonica* rice has resulted in a 10–30% improvement in nitrogen-use rate and yield compared with those of the control, indicating that *NRT1.1B* can enhance nitrogen-use efficiency in *japonica* rice (Hu et al. 2015). Nitrogen that is not absorbed by crops may enter the environment through nitrification, denitrification, volatilization, and runoff. When fertilization exceeds the carrying capacity of the soil, it can cause acidification and lead to the hardening and degradation of soils (Xu et al. 2012; Lai et al. 2022). Consequently, improving nitrogen-use efficiency would represent a key strategy for resolving these agricultural problems.

Microbial populations are closely associated with plant species, and *NRT1.1B* plays an important role in determining the composition of the rice root microbiota by promoting root microbial recruitment (Zhang et al. 2019a). The overall function of the plant microbial community is evidently greater than the sum of the functions of individual species, because microorganisms typically enter into symbiotic, parasitic, and synergistic interactions with plants, thereby forming complex networks and relationships (Miller et al. 2018; Rodriguez et al. 2019). These interactions stimulate plant growth, promote the synthesis of chemical substances, and influence plant health, soil fertility, and nutrient cycling, particularly the transformations of nitrogen and phosphorus (Stringlis et al. 2018; Pivato et al. 2021). The composition, diversity, and abundance of bacterial communities in the soil can serve as indicators of plant biomass, material cycling, and biological processes (Ju et al. 2020; Lai et al. 2022). These indicators are affected by both plants and external environmental factors, including the secretion of root exudates in the plant rhizosphere and the application of organic fertilizers, which can modulate microbial diversity, function, and nutrition content (Rojas et al. 2016; Hu et al. 2018).

Different rice varieties and genotypes are characterized by significant differences in nitrogen-use efficiency. Thus, fully exploiting the genetic potential of rice is considered an ideal approach for improving nitrogen-use efficiency (Li et al. 2014; Hu et al. 2015; Wang at al. 2018a). In this study, we investigated the effects of *NRT1.1B* expression and nitrogen fertilization on soil microbial community diversity and growth indices in rice. We hypothesized that *NRT1.1B* expression and nitrogen fertilization levels could (1) influence the rhizosphere soil bacterial diversity of rice and (2) change the growth indicators of rice plants. To the best of our knowledge, this is the first study to investigate the differences in rice soil microbial diversity and plant growth under different nitrogen application conditions and expression of nitrogen-use efficiency-related genes. Our findings will have important theoretical and practical implications for developing cultivars with higher nitrogen-use efficiency, optimizing nitrogen application, establishing targeted nitrogen application technology systems based on the variety, and enhancing the nitrogen fertilization efficiency of crops.

## Materials and methods

### Site description and experimental design

In May 2019, surface soil (0–15 cm) was collected from the farmland (119°01′25.21″ E, 33°31′25.33″ N) of a modern agricultural high-tech park in Huaian City, Jiangsu Province, China, using the five-point sampling method. The soil has a sandy loam texture, presents organic matter, total nitrogen, available phosphorus, and available potassium concentrations of 18.17, 1.33, 7.29, and 47.5 mg·kg^− 1^, respectively, and has a pH of 7.24. Seeds of the local rice cultivars *Huaidao 5* and *Xinhuai 5* were soaked in 3% (v/v) H_2_O_2_ solution for 1 h and raised in a seedling tray for 30 days. Subsequently, the seedlings of similar heights were selected and transplanted into planting buckets, with one plant per pot. The *NRT1.1B* gene was introduced into *Huaidao* 5 using molecular marker-assisted selection, and plants were cultivated as described above (Li and Li 2016).

### Experimental treatments and sampling

The recommended field standard of nitrogen is 150 kg N·ha^− 1^ (Adamu et al. 2015), and we established our study’s framework based on this standard for the following five treatments in this study: deionized water (control, 0 kg N·ha^− 1^), 100% (150 kg N·ha^− 1^), 75% (112.5 kg N·ha^− 1^), 50% (75 kg N·ha^− 1^), and 25% (37.5 kg N·ha^− 1^) nitrogen. Nitrogen was added at 50%, 10% and 40% of the base stage, tillering stage and booting stage respectively (Yang et al. 2020). Compound fertilizer is used as basic nitrogen fertilizer (N:P_2_O_5_:K_2_O = 15%:15%:15%), while the tillering and booting nitrogen fertilization was conducted with urea (CO(NH_2_)_2_, *N* ≥ 46.4%). Both the phosphate (P_2_O_5_) and potassium (K_2_O) fertilizers were only used as basal fertilizers. Dissolved nitrogen fertilizers were sprayed onto the soil surface. These treatments were applied to both the *Huaidao 5* (CK) and *Xinhuai 5* (XH) rice varieties, resulting in a total of 10 treatment combinations. Both varieties were grown in pots (diameter, 26 cm; height, 31 cm) filled with 15 kg sieved soil with four destructive samplings being performed at the tillering, booting, flowering, and maturity stages. Each treatment included three replicates, for a total of 120 pots. Pests, pathogens, and weeding were managed, when required, according to normal field management practices.

### Soil and biological properties

Additionally, the stems, leaves and ears of rice were harvested every growth period, sterilized indoors at 105 °C for 1 h, and then oven-dried at 60 °C for one week to a constant dry weight to measure the corresponding nitrogen content and total nitrogen content. The soil in which the rice plants were grown was carefully collected and stored in a refrigerator at 4 °C for subsequent analyses of soil microbial biomass, microbial community composition, pH, and enzyme activities. Rice roots were placed in 1-mm mesh bags, and the soil adhering to the surface was removed using running water. We immediately collected 0–5 cm root tip segments to determine root and endoenzyme activities.

### Chemical analyses and enzymatic assays

Root activity was determined using the triphenyl tetrazolium chloride method of Lindström and Nyström (1987). The activities of root enzymes involved in soil nitrogen cycling (e.g., nitrate reductase and urease) and metabolism in rice roots (e.g., nitrate reductase, glutamate synthetase, and glutamine synthetase) were determined spectrophotometrically. Enzyme activities were assayed within 1 week of sampling. Table S1 lists the conditions used in the enzymatic assays.

The dry matter mass of each part of rice plants was determined by weighing the dried plant materials. A SPAD-502 chlorophyll meter (Minolta, Osaka, Japan) was used to determine the chlorophyll status in rice. Soil and plant total nitrogen concentrations were determined by initially digesting the root samples with H_2_SO_4_–H_2_O_2_ at 60 ℃ and then measuring the concentrations using a San + + flow analyzer (Skalar, Breda, Netherlands).

### DNA extraction, amplification, and high-throughput sequencing

A HiPure Soil DNA kit (Magen, Guangzhou, China) was used for the extraction of total DNA from 0.5 g of soil following the manufacturer’s protocol. The target region (341 F: 5′-CCTACGGGNGGCWGCAG-3′; 806R: 5′-GGACTACHVGGGTATCTAAT-3′) of the 16 S ribosomal RNA (rRNA) gene was amplified using PCR (94 °C for 2 min, followed by 30 cycles at 98 °C for 10 s, 62 °C for 30 s, and 68 °C for 30 s, with a final extension at 68 °C for 5 min). Amplicons were extracted from 2% agarose gels, purified using an AxyPrep DNA Gel Extraction kit (Axygen Biosciences, Union City, CA, USA) following the manufacturer’s instructions, and quantified using the ABI StepOnePlus Real-Time PCR system (Life Technologies, Foster City, CA, USA). Purified amplicons were pooled in equimolar quantities and paired-end sequenced (PE250) with an Illumina platform using standard protocols. Subsequently, the results were submitted for Illumina pyrosequencing (https://www.ncbi.nlm.nih.gov/subs/ PRJNA974191) for sequence analysis.

### Statistical and bioinformatics analyses

The pyrophosphate sequencing results were assembled and filtered, and the primer regions were subsequently removed. The remaining sequences were assigned to operational taxonomic units (OTUs) using QIIME (version 1.9.1) based on a > 97% similarity using the UPARSE pipeline (version 9.2.64). The tag sequence with the highest abundance was selected as the representative sequence for each cluster. SPSS 22.0 (SPSS Inc., Chicago, IL, USA) was used to determine the differences in the bacterial community diversity in *NRT1.1B*-expressing rice soil and under different nitrogen application conditions using a two-way analysis of variance (ANOVA). The least significant difference test was used to determine whether the differences between the treatments were statistically significant. Statistical significance was set at *p* < 0.05. Chao1, Simpson, and other alpha diversity indices were calculated using QIIME. Rarefaction and rank-abundance curves were plotted using the R project ggplot2 package (version 2.2.1). We conducted a beta diversity analysis on the multivariate statistical data using a principal coordinate analysis of the weighted UniFrac, Jaccard, and Bray-Curtis distance values. The analysis was performed utilizing the Vegan package of the R platform and visualized by plotting with the ggplot2 package (version 2.2.1).

## Results

### Effects of nitrogen application and ***NRT1.1B*** expression on rice plant growth and yield

**Fig. 1:**
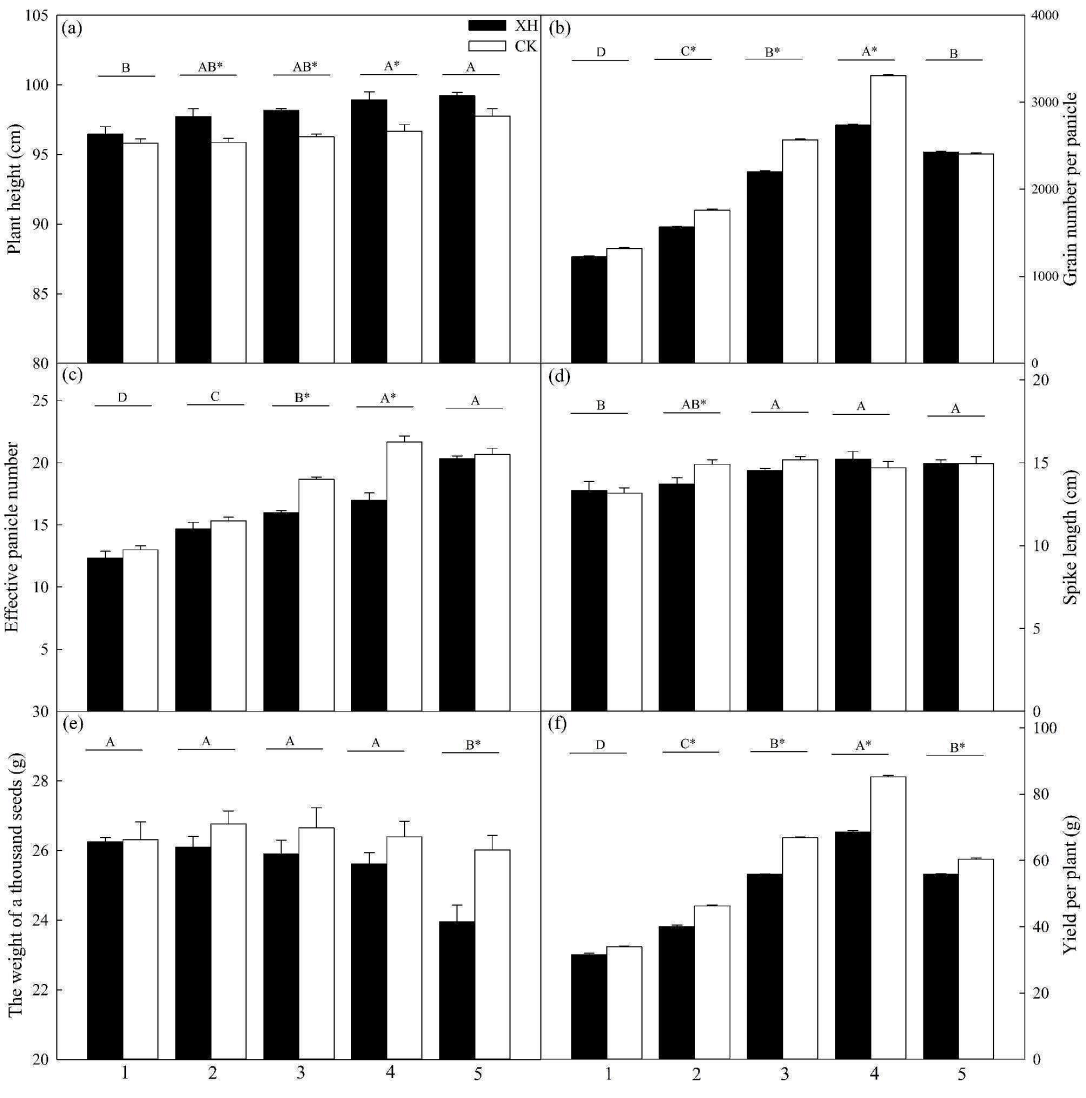
Effects of *NRT1.1B* expression and nitrogen application on plant growth indices and yield (*n* = 3): (**a**) plant height, (**b**) grain number per panicle, (**c**) effective panicle number, (**d**) spike length, (**e**) thousand-grain weight, and (**f**) yield per plant for the *Huaidao 5* and *Xinhuai 5* rice varieties treated with 1, 0% N; 2, 25% N; 3, 50% N; 4, 75% N; and 5, 100% N. Data are presented as the mean ± standard error (SE). *p < 0.05 between *Huaidao 5* and *Xinhuai 5* plants grown under the same nitrogen application (independent *t*-test). ^A, B, C, D^*p* < 0.05 among the corresponding nitrogen application and *NRT1.1B* expression conditions. Abbreviations: XH: *Xinhuai 5*; CK: *Huaidao 5*

In response to the application of nitrogen to *Huaidao 5* plants, we detected initial increases in plant height, grain number per panicle, spike length, and yield per plant, which then gradually plateaued. The highest values were observed in plants treated with the 75% nitrogen application. Additionally, under the assessed conditions, a gradual increase in the effective panicle number and a gradual reduction in thousand-grain weights were observed (Fig. 1). Similarly, we detected increases in the grain number per panicle, effective panicle number, spike length, thousand-grain weight, and yield per plant for *Xinhuai 5*, which then gradually plateaued; however, plant height gradually increased with an increase in nitrogen application (Fig. 1). We found that the expression of *NRT1.1B* contributed to a significant reduction (*p* < 0.05) in plant height after the application of 25, 50, and 75% nitrogen; however, significant increases were observed in spike length and yield per plant (*p* < 0.05) with 25% nitrogen application. The effective panicle number and grain number per panicle increased in the plants treated with 25, 50, and 75% nitrogen. Conversely, the thousand-grain weight increased in the plants treated with 100% nitrogen (Fig. 1).

**Fig. 2:**
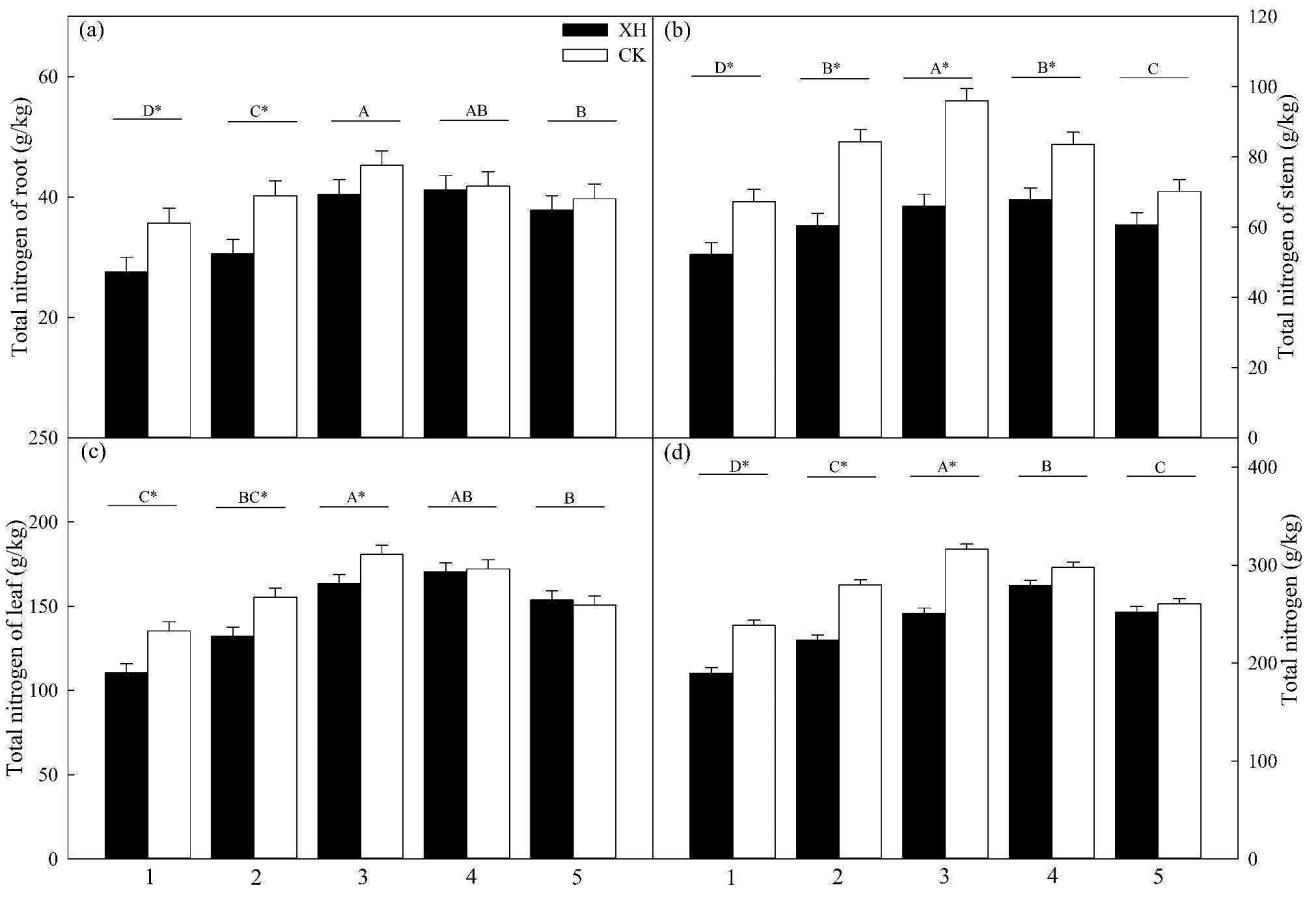
Effects of *NRT1.1B* expression and nitrogen application on rice root, stem, leaves, and total nitrogen content (*n* = 3). Treatments: 1, 0% N; 2, 25% N; 3, 50% N; 4, 75% N; and 5, 100% N. Data are presented as the means ± SE. *p < 0.05 between *Huaidao 5* and *Xinhuai 5* plants under the same nitrogen application conditions (independent *t*-test). ^A, B, C, D^*p* < 0.05 among the corresponding nitrogen application and *NRT1.1B* expression conditions. Abbreviations: XH: *Xinhuai 5*; CK: *Huaidao 5*

After nitrogen application, we observed an initial increase in the nitrogen contents of *Huaidao 5* and *Xinhuai 5* rice roots, stems, and leaves, which subsequently declined. The highest values were attained in the 50 and 75% nitrogen application groups (Fig. 2). *NRT1.1B* expression also contributed to significant increases in the nitrogen contents of roots after 0 and 25% nitrogen application. Additionally, increases in leaves occurred after 0, 25, and 50% application, and in the stems after 0, 25, 50, and 75% application (*p* < 0.05; Fig. 2). Furthermore, we observed an initial increase in the nitrogen fertilizer and agricultural nitrogen fertilizer-use efficiencies of *Huaidao 5* followed by a subsequent sharp decline. In contrast, the nitrogen fertilizer- and agricultural nitrogen fertilizer-use efficiencies of *Xinhuai 5* showed a significant increase following treatment (*p* < 0.05; Table 1). Moreover, rice expressing *NRT1.1B* showed nitrogen fertilizer and agricultural nitrogen fertilizer efficiencies compared to the untreated controls (*p* < 0.05; Table 1).

**Table 1:** Nitrogen fertilizer absorption and use rate and nitrogen-use efficiency of rice expressing *NRT1.1B* grown under different nitrogen treatment conditions

Type	Treatments	Nitrogen fertilizer use efficiency	Agronomic nitrogen fertilizer use efficiency
XH	1	0 c	0 c
	2	2.32 b	1.54 b
	3	11.46 a	6.12 a
	4	17.31 a	9.56 a
	5	9.02 ab	4.57 ab
CK	1	0 c	0 c
	2	15.48 b	7.23 b
	3	18.91 b	9.61 ab
	4	22.29 ab	11.19 a
	5	26.69 a	9.53 ab

Treatments: 1, 0% N; 2, 25% N; 3, 50% N; 4, 75% N; and 5, 100% N (*n* = 3). ^a, b, c^*p* < 0.05 among corresponding nitrogen application rates and *NRT1.1B* expression conditions. Abbreviations: XH: *Xinhuai 5*; CK: *Huaidao 5*

**Fig. 3:**
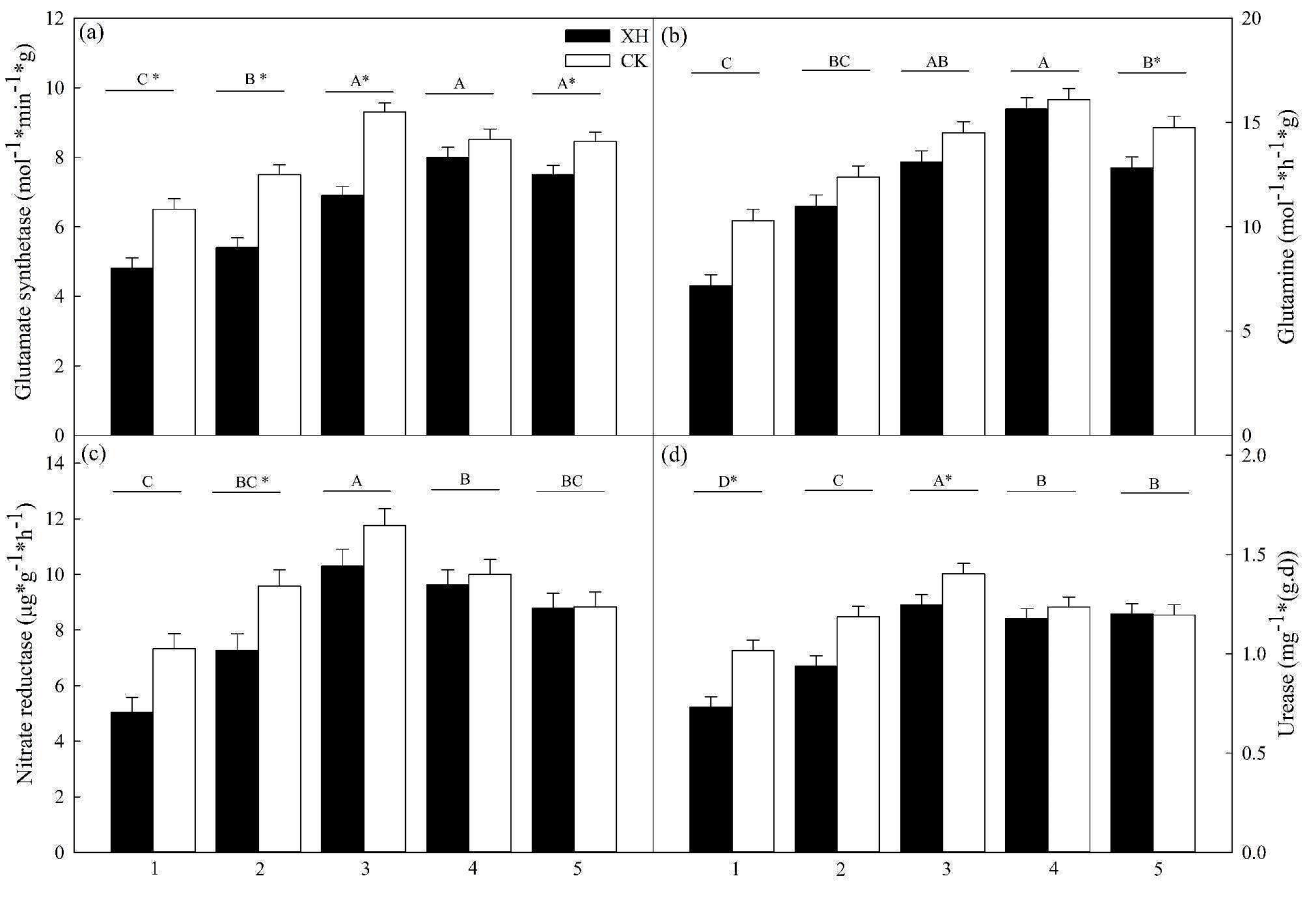
Effects of *NRT1.1B* expression on enzyme activities in rice seedlings grown under different nitrogen conditions (*n* = 3): (**a**) Glutamate synthetase, (**b**) glutamine, (**c**) nitrate reductase, and (**d**) urease activities in rice grown with 1, 0% N; 2, 25% N; 3, 50% N; 4, 75% N; and 5, 100% N. Data are presented as the means ± SE. *p < 0.05 between *Huaidao 5* and *Xinhuai 5* plants grown under the same nitrogen application conditions (independent *t*-test). ^A, B, C, D^*p* < 0.05 among the corresponding nitrogen application and *NRT1.1B* expression conditions. Abbreviations: XH: *Xinhuai 5*; CK: *Huaidao 5*

The glutamate synthetase levels in *Xinhuai 5* initially increased and then decreased in response to nitrogen application, reaching the highest values after 50% nitrogen application; however, in *Huaidao 5*, the highest values were observed in response to the 75% nitrogen treatment. *NRT1.1B* expression promoted a significant increase in the glutamate synthetase content of rice in all other groups (*p* < 0.05; Fig. 3a), except the 75% nitrogen application group. Glutamine synthase contents in *Huaidao 5* and *Xinhuai 5* initially increased and then decreased in response to nitrogen application, reaching the highest values at 75% application; however, *NRT1.1B* expression contributed to a significant increase in the glutamine synthase content in plants at 100% application (*p* < 0.05; Fig. 3b). The contents of nitrate reductase and urease in *Huaidao 5* and *Xinhuai 5* also initially increased and subsequently decreased in response to nitrogen application, reaching the highest values in the 50% nitrogen treatment group. Among these plants, *NRT1.1B* promoted a significant increase in nitrate reductase and urease in those treated with 25 and 50% nitrogen (*p* < 0.05; Fig. 3c and d).

**Fig. 4:**
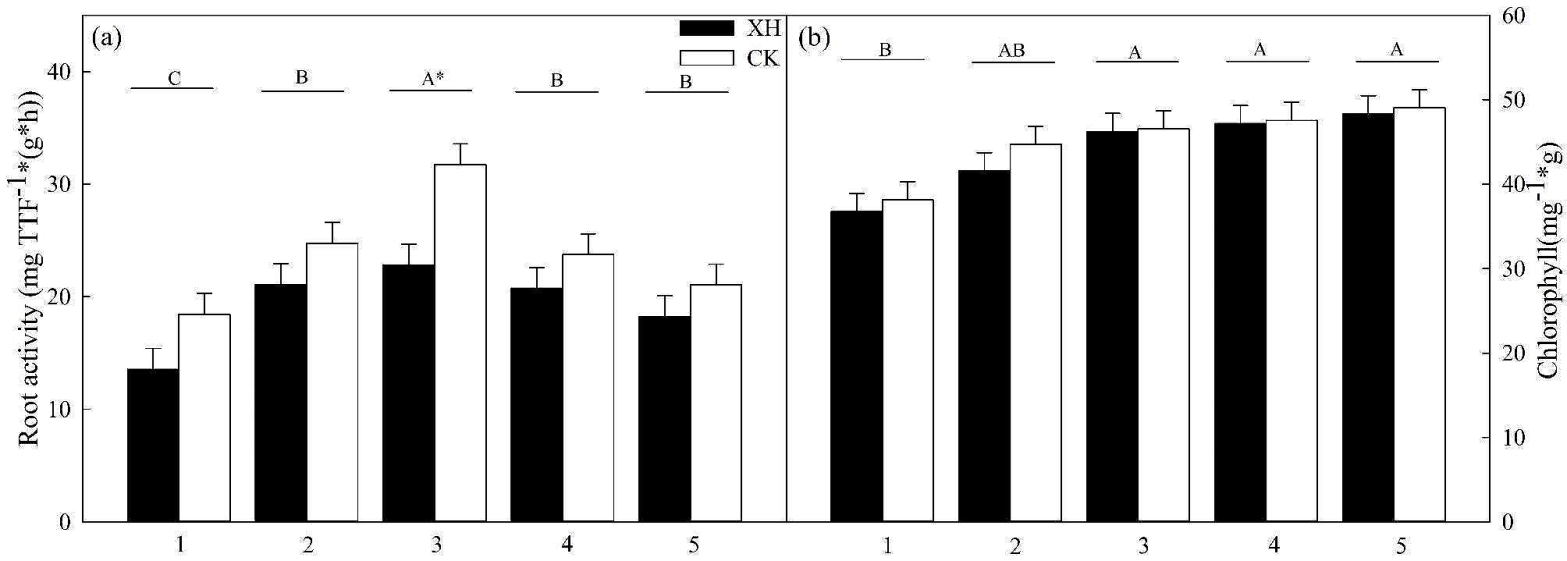
Effects of *NRT1.1B* expression and nitrogen application on (**a**) root activity and (**b**) chlorophyll content (*n* = 3). Treatments: 1, 0% N; 2, 25% N; 3, 50% N; 4, 75% N; and 5, 100% N. Data are presented as the means ± SE. *p < 0.05 between *Huaidao 5* and *Xinhuai 5* plants under the same nitrogen application conditions (independent *t*-test). ^A, B, C^*p* < 0.05 among the corresponding nitrogen application and *NRT1.1B* expression conditions. Abbreviations: XH: *Xinhuai 5*; CK: *Huaidao 5*

The root activities of *Huaidao 5* and *Xinhuai 5* rice initially increased and subsequently declined after nitrogen treatment, reaching the highest values at 50% nitrogen application. At this application level, *NRT1.1B* expression led to a significant increase in root activity (*p* < 0.05; Fig. 4a). Moreover, we detected gradual increases in chlorophyll levels in *Huaidao 5* and *Xinhuai 5* rice in the 50% nitrogen treatment group, which subsequently plateaued. The expression of *NRT1.1* similarly contributed to an increase in chlorophyll levels, although the effect was non-significant (Fig. 4b).

### Effects of nitrogen application and ***NRT1.1B*** expression on bacterial community composition in rice soil

**Fig. 5:**
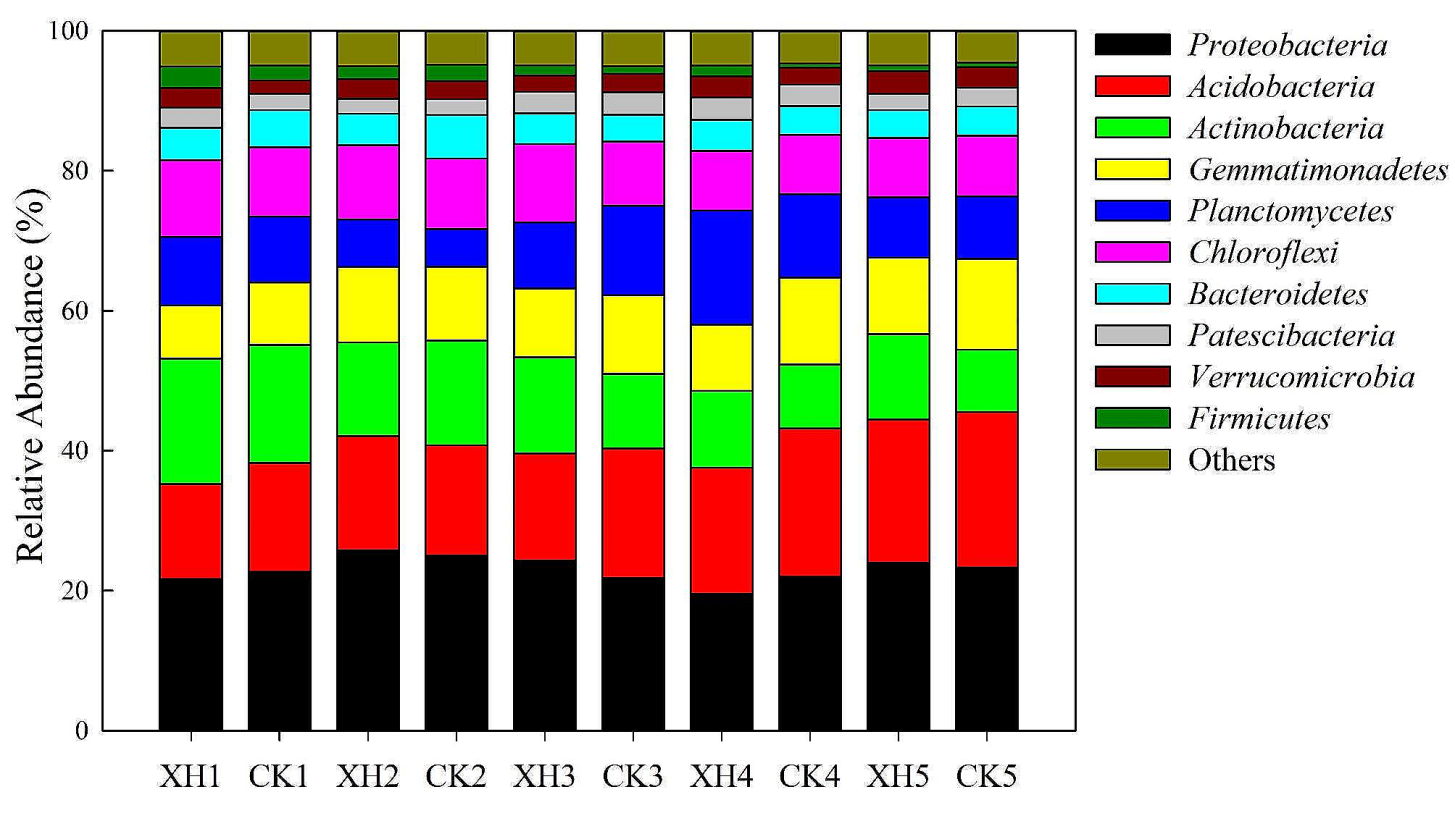
Effects of *NRT1.1B* expression and nitrogen application on relative bacterial abundance at the phylum level in rice soil (*n* = 3). Treatments: 1, 0% N; 2, 25% N; 3, 50% N; 4, 75% N; and 5, 100% N. Abbreviations: XH: *Xinhuai 5*; CK: *Huaidao 5*

**Fig. 6:**
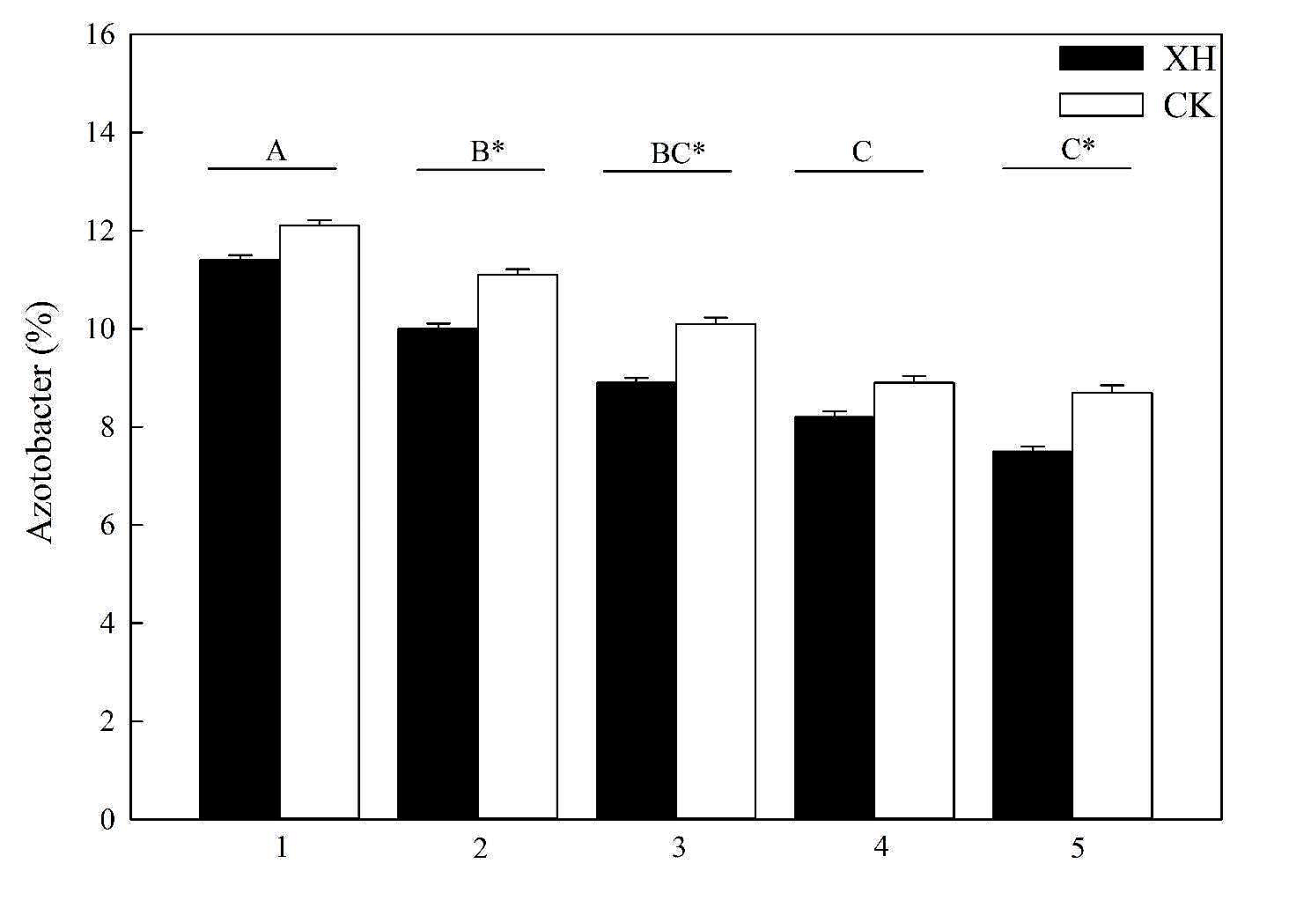
Effects of *NRT1.1B* expression and nitrogen application on the proportion of nitrogen-fixing bacteria at the genus level in rice soil (*n* = 3). Treatments: 1, 0% N; 2, 25% N; 3, 50% N; 4, 75% N; and 5, 100% N. Data are presented as the means ± SE. *p < 0.05 between *Huaidao* 5 and *Xinhuai* 5 plants under the same nitrogen application conditions (independent *t*-test). ^A, B, C^*p* < 0.05 among the corresponding nitrogen application and *NRT1.1B* expression conditions. Abbreviations: XH: *Xinhuai 5*; CK: *Huaidao 5*

To assess the effects of *NRT1.1B* expression and nitrogen application on soil microbiota, we compared the structures of the soil microbial communities associated with the two rice varieties. In total, we detected 103,463 and 98,427 OTUs in the soils used to cultivate *Huaidao 5* and *Xinhuai 5* rice, respectively. In both crop fields, *Proteobacteria*, *Acidobacteria*, *Actinobacteria*, *Gemmatimonadetes*, *Planctomycetes*, and *Chloroflexi* were identified as the most abundant microbial phyla (Fig. 5). We established that *NRT1.1B* expression and nitrogen application promoted significant changes in the composition of the rice soil bacterial community (*p* < 0.05; Fig. 5). In response to an increase in nitrogen application, we detected a significant reduction in the number of *Azotobacter* spp. in *Huaidao 5* and *Xinhuai 5* rice soil (*p* < 0.05; Fig. 6); however, *NRT1.1B* expression contributed to a significant increase in the proportion of nitrogen-fixing bacteria at the genus level in the rice soil receiving 25, 50, and 100 nitrogen inputs (*p* < 0.05; Fig. 6). The rarefaction curve analysis, which was based on a 97% identity cutoff for 16 S rRNA gene sequencing in rice soil, supported the adequacy of our sample sequencing data for microbial community diversity assessment. The depth of sequencing, coupled with considerations of feasibility and rationality, indicated that acquiring additional data was unnecessary to perform meaningful analyses (Fig. S1). *NRT1.1B* expression, nitrogen application, and their interactions had significant effects on the bacterial communities in the *Huaidao 5* and *Xinhuai 5* rice soils (*p* < 0.05; Table 2).

**Table 2:** The statistical test of similarity (ANOSIM) and permutational multivariate two-way analysis of variance (PERMANOVA) to analyze the effect of *NRT1.1B* and nitrogen application on bacterial community composition calculated by Illumina sequencing

Type	Treatment		df	PERMANOVA		ANOSIM	
Soil	*NRT1.1B*		1	7.5342	0.0045	0.7824	0.0271
	Nitrogen application		4	3.4694	0.0061	0.6425	0.0034
	*NRT1.1B* X Nitrogen application		4	5.2145	0.0417	0.4287	0.0057

### α and β diversity of soil bacterial communities

**Fig. 7:**
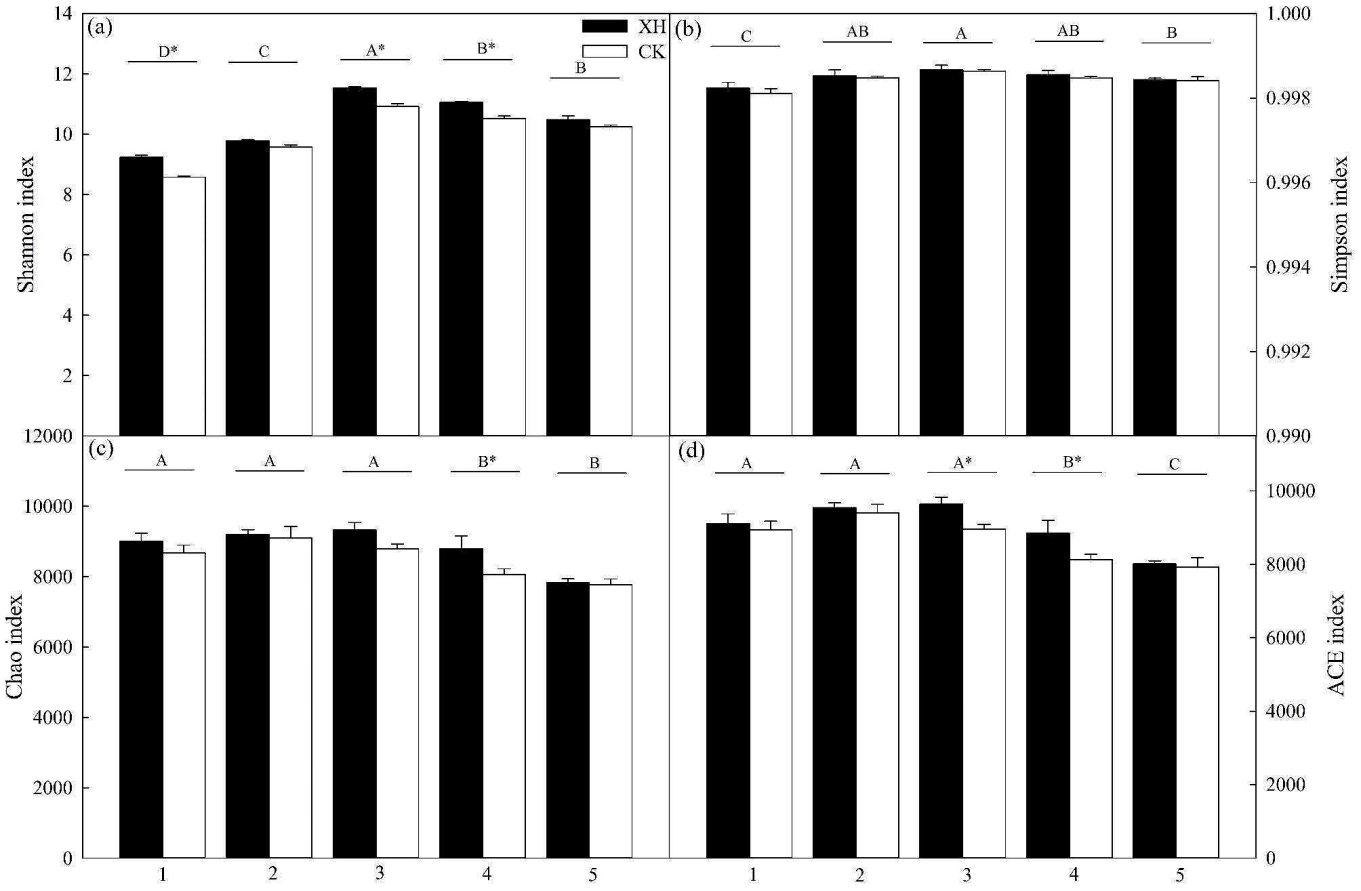
Soil bacterial diversity and richness are affected by *NRT1.1B* expression and nitrogen application (*n* = 3). Treatments: 1, 0% N; 2, 25% N; 3, 50% N; 4, 75% N; and 5, 100% N. Data are presented as the means ± SE. *p < 0.05 between *Huaidao 5* and *Xinhuai 5* plants under the same nitrogen application conditions (independent *t*-test). ^A, B, C, D^*p* < 0.05 among the corresponding nitrogen application and *NRT1.1B* expression conditions. Abbreviations: XH: *Xinhuai 5*; CK: *Huaidao 5*

Our study revealed substantial impacts of nitrogen application and *NRT1.1B* expression on the diversity and richness of bacterial communities in the *Huaidao 5* and *Xinhuai 5* rice soils (Fig. 7). Nitrogen application initially led to increases in the Shannon and Simpson indices, followed by a subsequent decline; however, it resulted in reductions in both the Chao and ACE indices (Fig. 7).

**Fig. 8:**
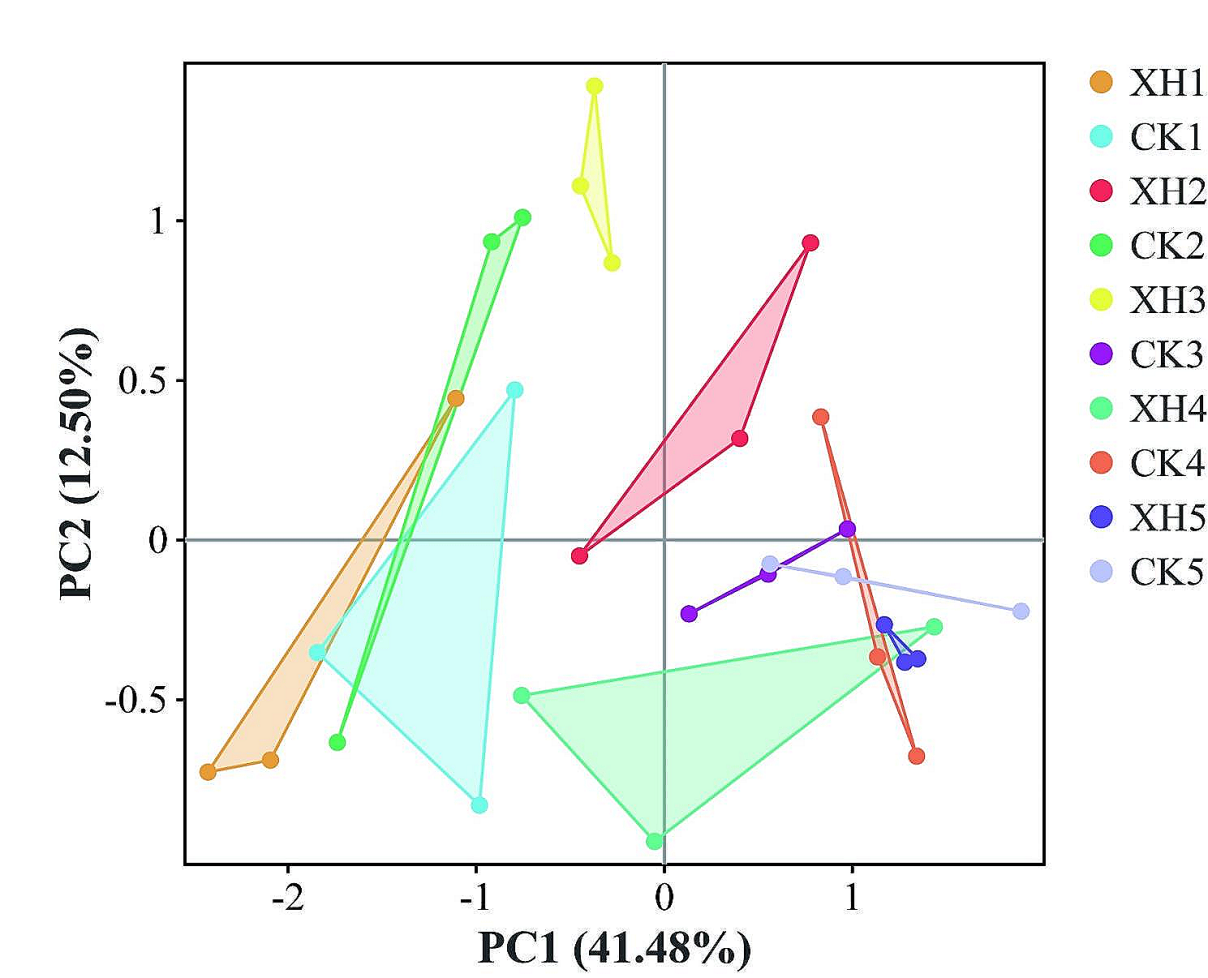
Rice soil bacterial community structure. Principal coordinate analysis of the pairwise Bray–Curtis dissimilarity within each of the three datasets tested with randomly sampled soil microbial communities. Abbreviations: XH: *Xinhuai 5*; CK: *Huaidao 5*

These findings accordingly indicate that the bacterial diversity of rice soil initially increased and subsequently declined after fertilization, peaking at the highest value in response to 50% nitrogen application. However, we observed a significant reduction in rice soil bacterial richness occurred with an increase in the level of applied nitrogen (*p* < 0.05; Fig. 7). Furthermore, *NRT1.1B* expression resulted in a significant reduction in the bacterial diversity in soils treated with 0, 50, and 75% nitrogen, and reductions in the bacterial richness of soil treated with 50 and 75% nitrogen (*p* < 0.05; Fig. 7). Principal coordinate analysis and permutational multivariate ANOVA revealed that *NRT1.1B* expression and nitrogen application significantly altered the composition of the microbial communities of rice soil, thus leading to differences in the community structure (Fig. 8; Table 2).

## Discussion

Although the application of large amounts of nitrogen fertilizer can contribute to high rice yields, nitrogen-use efficiency is a major factor limiting rice production (Fang et al. 2012; Fu et al. 2023). A potentially effective strategy for enhancing rice productivity is to substantially increase plant nitrogen-use efficiency (Fang et al. 2012; Wang et al. 2018a; Fu et al. 2023). Plant diversity and associated microbial communities can either directly or indirectly determine nitrogen absorption and nitrogen-use efficiency in plants, thereby serving as reliable indicators of plant growth, development, and nutrient metabolism. Consequently, in this study, we aimed to determine the effects of *NRT1.1B* expression and nitrogen fertilization on the rice soil microbial diversity and growth indices. We found that both soil microbial diversity and plant growth indices initially increased and then decreased in response to an increase in the amount of applied nitrogen fertilizer. These findings reveal that the reduction in nitrogen-use efficiency was associated with excessive nitrogen application, which is consistent with previously reported findings (Sun et al. 2012; Fu et al. 2023).

### Effects of ***NRT1.1B*** expression and nitrogen application on rice growth, enzyme activities, and other indicators

Pan et al. (2016) have previously observed significant positive correlations between nitrogen accumulation and rice root surface area, total length, and volume; however, Ju et al. (2015) have reported differences in crop yield and nitrogen uptake among different rice genotypes and nitrogen supply level interactions under different irrigation conditions. Ju et al. (2015) suggested that the high nitrogen uptake efficiency of two *japonica* rice varieties can be attributed to long root length and high root oxidation activity. Similarly, in the present study, we demonstrated that the application of nitrogen fertilizer can contribute to promoting rice growth and plant yield (Figs. 1 and 2). At equivalent levels of nitrogen fertilizer application, rice varieties demonstrating a high nitrogen-use efficiency exhibited a 38% higher yield than those demonstrating a low nitrogen-use efficiency. This response highlights the positive impact of nitrogen fertilizer application on crop yield (Gueye et al. 2011; Li et al. 2012). In our study, the application of nitrogen fertilizer promoted the effective absorption and accumulation of nitrogen in rice plants (Table 1). Furthermore, the expression of *NRT1.1B* in rice plants plays a role in mediating the transport of selenomethionine from roots to stems (Zhang et al. 2019b). Hu et al. (2019) also has found that nitrate-*NRT1.1B*-SPX4 cascade integrates nitrogen and phosphorus signalling networks in plants. We observed that the *NRT1.1B* expression promoted significant increases in tiller number, stem dry weight, leaf dry weight, and root activity in rice plants concomitant with an increase in nitrogen fertilizer treatment (Fig. 6). We hypothesize that this effect could be attributed to the role of *NRT1.1B* in enhancing nitrate uptake and root-to-shoot transport via the upregulation of nitrate-related gene expression, which significantly enhanced rice yield and nitrogen-use efficiency (Hu et al. 2015). In addition, nitrogen-efficient rice varieties exhibited significant advantages over their inefficient counterparts, which were characterized by high root biomass, volume, total absorption surface area, and active absorption area (Gueye et al. 2011; Li et al. 2012; Hu et al. 2015).

The plant root systems release a diverse array of compounds, known as root exudates, that play crucial roles in feedback mechanisms. These exudates regulate the growth and development of the root system, along with the absorption and use of nitrogen, phosphorus, and potassium (Li et al. 2014). In rice soil, the conversion of NO_3_^−^ and NH_4_^+^ involves various ammonium salt transporters that are closely coordinated with enzymes such as nitrate reductase, nitrite reductase, glutamine synthetase, and glutamate synthetase, that can influence rice plant growth and, consequently, crop yield (Krapp et al. 2005; Tabuchi et al. 2007). Our findings further revealed that the expression of *NRT1.1B* can promote significant increases in the contents of glutamic acid, glutamine synthetase, nitrate reductase, and urease in rice soil (Fig. 3), This suggests a potential mechanism through which *NRT1.1B* alters nitrogen levels in rice soil.

### ***NRT1.1B*** expression and nitrogen application influence rice soil bacterial communities

Our analysis revealed that the bacterial community in soil used to cultivate rice primarily comprised *Proteobacteria* and *Acidobacteria*, which is consistent with the findings of previous studies on the bacterial diversity associated with rice. Proteobacteria, Acidobacteria, Actinobacteria, and Gemmatimonadetes have consistently been identified as the dominant microbial phyla in rice soils (Edwards et al. 2015; Shenton et al. 2016). Furthermore, Liu et al. (2021) have shown that the interaction between eCO_2_ and nitrogen fertilizer not only promoted a significant increase in the relative abundance of *Methylococcus* but also significantly reduced the relative content of *Rhizobiaceae*. In general, nitrogen inputs tend to inhibit its bacterial nitrogen fixation, given the sensitivity of the *nifH*-encoded nitrogenase to nitrogen concentrations (Smercina et al. 2019). The results obtained in the present study indicated that the application of nitrogen reduces the abundance of *Azotobacter* spp. and induces changes in the diversity and richness of bacteria and other microorganisms (Figs. 6 and 7, and 8). In this context, Edwards et al. (2015) revealed genotypic differences in the rhizosphere communities of *indica* and *japonica* rice subspecies. Our investigation further revealed that the expression of *NRT1.1B* leads to the formation of different clusters of rice-associated microbes (Figs. 5 and 7), a phenomenon similar to that observed in previous studies (Zhang et al. 2019a). We speculate that *NRT1.1B* contributed to altering the composition, diversity, and richness of microbial communities in rice soil by improving the nitrogen absorption capacity of rice plants compared with the control; thus, microbial enrichment or external nitrogen sources were not required (Fig. 7).

Consequently, we posit that the expression of *NRT1.1B* and application of nitrogen exert an influence by reducing microbial diversity and richness in rice soil. As described by Zhang et al. (2019a), *NRT1.1B* contributes to the enrichment and establishment of the bacterial flora in rice roots and promotes the differentiation of root microbiota among different rice varieties. These effects are closely associated with the abundance of key genes involved in the nitrogen cycle, which play crucial roles in the formation of ammonium compounds in plant roots. Our findings are consistent with previously reported findings and show that *NRT1.1B* can promote significant increases in the proportion of *Azotobacter*, thereby enhancing nitrogen absorption in the two assessed rice varieties. This is further supported by high nitrogen contents observed in the roots, stems, and leaves of plants expressing *NRT1.1B* (Figs. 2 and 7). Similarly, *AtNRT2.1*, *AtNRT2.2*, and *AtNAR2.1* have been identified as important contributors to the high-affinity nitrate transport system in *Arabidopsis* roots (Orsel et al. 2006). These results collectively indicate that nitrogen-use efficiency-related genes can contribute to enhancing the absorption and transformation of nitrogen and regulate plant growth in rice (Feng et al. 2011). However, the mechanism by which the *NRT1.1B* gene effectively modulates rice root microbial communities and plant growth under different conditions of nitrogen application has not been clarified. Accordingly, further experiments are needed to clarify these underlying mechanisms, which will contribute to significantly enhancing the nitrogen-use efficiency of rice.

In this study, under nitrogen application, an initial increase and subsequent decrease in bacterial diversity and a linear reduction in microbial richness were observed in the *Huaidao 5* and *Xinhuai 5* rice soils. Compared with the diversity and abundance of bacteria in the soil of *Xinhuai 5* rice, those in the soil of *Huaidao* 5 rice were reduced in response to the application of nitrogen, which also had the effect of reducing the number of nitrogen-fixing bacteria. Moreover, the expression of *NRT1.1* enhanced the rate of nitrogen absorption rate in *Xinhuai 5* rice by promoting the aggregation of nitrogen-fixing bacteria and increasing root activity. This enhancement was evident through increases in tiller number, yield, and income under low-nitrogen conditions. Our findings provide insights into the role of *NRT1.1B* in enhancing the effective use of nitrogen fertilizers for rice production and establishing a technical system for efficient nitrogen fertilizer application. Future studies should focus on systematically characterizing the mechanisms underlying the effects of nitrogen fertilizer application and *NRT1.1B* expression on rice root bacterial communities and plant growth.

### Electronic supplementary material

Below is the link to the electronic supplementary material.

Supplementary Material 1

## Data Availability

The data that support the findings of this study are available in tables and figures.
