# Supplementary material for: NRT1.1B mediates rice plant growth and soil microbial diversity under different nitrogen conditions
Source: AMB Express. 2024 Apr 22;14:39. doi: 10.1186/s13568-024-01683-7 (PMC11035536; doi:10.1186/s13568-024-01683-7)
Supplement: Supplementary file 1 — Supplementary Material 1 [file 13568_2024_1683_MOESM1_ESM.docx]

**Table S1** Enzyme activities and assayed in soil and root, their corresponding substrates, active international unit (IU) definition, and references in the sodium subsidies experiment.

| Enzyme | Substrate | IU Definition |
| --- | --- | --- |
| glutamate synthetase | glutamate | 1 μmol glutamylmonohydroxamate released min^−1^ g^−1^ root |
| glutamine synthetase | glutamine | 1 μmol 2-ketoglutarate required min^−1^ g^−1^ root |
| Nitrate reductase | potassium nitrate | 1 μg NO_2_^−^ released min^−1^ g^−1^ soil |
| Urease | Urea | 1 mg NH_3_−N released h^−1^ g^−1^ soil |





Figure S1 Rarefaction curves of the bacterial 16S rRNA gene sequence from soil under rice expressing *NRT1.1B* and grown under different nitrogen conditions (n = 3). Treatments: 1, 0% N; 2, 25% N; 3, 50% N; 4, 75% N; and 5, 100% N. Abbreviations: XH, *Xinhuai* 5; CK, *Huaidao* 5.
